# Socioeconomic Inequalities in Adolescent Depression in South Korea: A Multilevel Analysis

**DOI:** 10.1371/journal.pone.0047025

**Published:** 2012-10-15

**Authors:** Hye Yin Park, Jongho Heo, S. V. Subramanian, Ichiro Kawachi, Juhwan Oh

**Affiliations:** 1 Department of Preventive Medicine, Seoul National University College of Medicine, Seoul, Republic of Korea; 2 Center for Health Equity Research and Policy, San Diego State University, San Diego, California, United States of America; 3 Department of Society, Human Development, and Health, Harvard School of Public Health, Boston, Massachusetts, United States of America; 4 Institute of Health Policy and Management, Medical Research Center, Seoul National University, Seoul, Republic of Korea; Université Catholique de Louvain, Belgium

## Abstract

**Background:**

In recent years, South Korea has witnessed a sustained rise in the prevalence of adolescent depression. In the present study, we sought to investigate family and school environmental influences on adolescent depression.

**Methods and Findings:**

Middle and high school students (N = 75,066) were randomly selected respondents to a web-based survey and answered questions on their academic and socioeconomic backgrounds, parental support, parental education level, physical activities, lifestyle habits and their experience of depression in the past one year. Two-level multilevel analysis was used to investigate the relationship between depression and individual (level 1) and school (level 2) factors. Girls reported having experienced depression in greater numbers than boys (43.96% vs. 32.03%). A significant association was found between adolescent depression experience and gender, grade, self-rated academic achievement, family affluence scale, parental support, parental education level, lifestyle habits, physical activity and sleep dissatisfaction. The students living with rich parents were more likely to be depressive, and maternal higher education was significantly associated with higher probability of boys’ depression experience. Low academic achievement was highly associated with the experience of depression. In school level contexts, girls were found to be less likely to be depressive in girls-only schools.

**Conclusion:**

The adolescent depression experience is not only an individual phenomenon but is highly associated with other factors such as parents, peers, academic achievement, and even gender mix in the school. Thus, prevention measures on youth depression need to focus on emphasizing less pressure from parents on academic performance, and establishing healthy inter-gender relationships within co-education schools.

## Introduction

Adolescent mental health has drawn growing attention in South Korea due to a sustained increase in adolescent depression as well as suicide incidence. The lifetime prevalence for adolescent depression has been reported to be as high as 20% [1], and 90% of adolescent suicides have mental health problems with depression as a major contributor [2].

Although there is no report on the prevalence of adolescent major depression disorder (MDD) [3], some clinical research teams and government organizations have attempted to investigate the prevalence and possible risk factors for adolescent depression using different assessment approaches in Korea. In 2005, a survey on adolescents residing in Seoul found that while depression prevalence based on parental report was only 0.86%, the estimated prevalence by self-report was almost ten times higher, 7.37% [4]. Considering the potential under-estimation even by self-report, the researchers speculated that the actual prevalence of adolescent depression could be as high as 9–13% [4]. In fact, another national-level survey in 2007 disclosed that 12% of students endorsed having “experienced depression” [5].

Reported risk factors for depression include being female, anxious, offspring of depressed parents, having subclinical levels of depressive symptoms, and being exposed to stress or trauma [6]. Yet many of the known contributing factors have been described in the western context, and it is not known how applicable they are to South Korean adolescents. Previous epidemiological and clinical research has indicated that adolescents are psychologically vulnerable to the influence of their social surroundings [7].

## Methods

### Source of Data

Data was used from The Fifth Korean Youth Risk Behavior Web-based Survey (KYRBWS), a national representative cross-sectional study with 76,937 students (7^th^ to 12^th^ school grade), from 800 middle and high schools in 2009. The survey was conducted by Korea Centers for Disease Control and Prevention (KCDC), which is legally approved agency to produce a national representative statistics based on The Statistical Act ensuring the quality of data and ethical consideration as well (http://yhs.cdc.go.kr). The KCDC used a stratified, two-stage cluster sampling. Four hundred middle schools and four hundred high schools were selected nationwide (first stage), and 3 classes from each school were sampled (second stage). Students filled out an online questionnaire which guaranteed anonymity. The response rate of the Fifth KYRBWS study was 97.6%. After excluding students with missing body mass index (BMI) values [8], 75,066 (2.4%) were selected as the total study population.

### Definition of Variables

#### Outcome variables

As the KYRBWS was a self-answered questionnaire on various fields, there were no specific questions for clinically diagnosing adolescent depression. Instead, the outcome variable ‘depression experience’ was used from responses to the question, “In the recent 12 months, have you experienced sadness or despair that interrupted your everyday life throughout two weeks’ time?” Answering yes or no to the single question was coded as the binomial variable. Although this question may not apply for clinically diagnosed depression, it is equivalent to the ‘two questions method’ (except duration: one month) used in screening depression in primary medical care [9].

There was no missing data to this variable, as all the respondents (100%) fulfilled answering to the depression experience question.

#### Individual-Level variables

Individual level variables included socio-demographic characteristics, health behaviors and physical factors of students as well as parental socio-economic status (SES). Self-rated academic achievement was a five category-variable, from very high to very low. Parental support was categorized into three categories: living with both of parents (including stepparents), single parent, or neither of the parents. Parental education level and Family Affluence Scale (FAS) were used to assess parental SES. Paternal and maternal education attainments were categorized into three categories, respectively: 1) middle school or less; 2) high school; 3) college or more. The Family Affluence Scale, which was developed by the European Health Behavior in School-aged Children study (HBSC), was adapted to measure family economic status, which cannot be accurately derived from children and adolescents (http://www.hbsc.org). The FAS is based on four questions: 1) having one’s own bedroom; 2) number of family trips per year; 3) total number of computers at home; and 4) number of vehicles owned by family. Smoking and alcohol drinking habits were measured as number of cigarettes and average volume of alcohol drinking per day in recent 30 days. Substance abuse was divided into three categories (never used / past experience of substance abuse / currently in abuse). To assess the status of internet addiction, the “Internet Addiction Proneness Scale-Short Form (KS scale)” was used [10]. It consists of 20 questions with 4 scales, from “never” to “always”, to differentiate addiction status: “normal”, “latent risk”, and “high risk.” Strenuous and moderate physical activities were defined as “number of days of strenuous exercise over 20 minutes” and “number of days of moderate exercise over 30 minutes”, respectively. Self-rated health and sleep satisfaction were grouped into 5 categories from very well to very poor. BMI was treated as a continuous variable.

#### School-Level contextual variables

Two types of school-level characteristics were used in the analysis. Urbanicity of the school location was classified as metropolis, cities, or rural area, and schools were likewise characterized as co-education, boys-only, or girls-only school.

### Statistical Analysis

Distribution of depression experience according to various individual and school characteristics was investigated. A two-level multilevel logistic regression model was applied for estimating differential effects of various socio-demographic factors, school performance, life-style, psychological, and school contextual characteristics on all students and on male and female students separately.

Once maximal likelihood estimates were obtained for starting values of the distribution, we ran random intercept models with Markov Chain Monte Carlo (MCMC) function by Bayesian approach, using MLwiN (development version 2.22). The MCMC simulates values in two-stage process (500 ‘burn-ins’ to discard, and 5,000 further simulations to get precise estimate) to reach to the estimate and distribution of interest. Empirical summaries of these simulated values are attained and convergence diagnostics is confirmed, which for ease of interpretation, is presented in odds ratios (OR) and 95% credible intervals (CI) in our study.

In model 1, student gender, school grades, self-rated academic achievement, the FAS, parental support, alcohol drinking and smoking volumes, substance abuse, internet addiction, high and moderate level physical activity, weight training, self-rated sleep satisfaction, and health status as individual level variables, and schools’ gender type (co-education / boys-only / girls-only school), and urbanicity of the school location served as school-level variables. As we assumed that there was no influence of parental education level on students not living with the parent/parents, we excluded them (N = 1,538) in model 2 and substituted parental support with parental educational attainment. Thus, we were able to examine the association between depression experience and parental support in model 1, and parental educational attainment variable in model 2.

## Results

Table 1 shows depression prevalence according to various individual and school predictors. Female students were shown to have a higher prevalence of depression than male students (43.96% vs. 32.03%). The students who were more likely to experience depression were those who were in higher school grades and of low academic achievement. Students who lived with a single parent or no parent experienced more depression than students living with both. Students who had parents with middle school or less educational attainment showed the highest response rates of depression (41.66% in fathers and 41.08% in mothers) compared with other education attainments. Students who did not live with parent(s) did not respond to the question on parental educational attainment (N = 6,856; 99.9%). Also, a large percentage of students living with parents or parent answered that they did not know their parents’ education attainment (N = 18,890; 13.3%). Of these, 32.7% answered to have depression experience, dissimilar to those who answered their parents’ education level. There was no difference in the FAS between those having and not having experienced depression. Students with unhealthy habits of smoking, drinking, substance abuse, and internet addiction were more likely to have experienced depression. Differences were observed on aspects of physical activities, self-rated sleep satisfaction and health status, but BMI showed little difference among the groups with and without depression experience.

**Table 1:** Characteristics of study subjects by ‘depression’ status.

		Depression N (%)	
		No	Yes
Gender	male	26926(67.97)	12686(32.03)
	female	19867(56.04)	15587(43.96)
Self-rated academic achievement	very low	5202(53.55)	4512(46.45)
	low	11469(59.93)	7669(40.07)
	middle	12786(63.24)	7433(36.76)
	high	11619(66.08)	5964(33.92)
	very high	5717(67.96)	2695(32.04)
Parental support	both parents	43821(62.83)	25920(37.17)
	single parent	2174(57.41)	1613(42.59)
	none	798(51.89)	740(48.11)
Paternal education	Middle or less	3028(58.34)	2162(41.66)
	High	17682(62.68)	10527(37.32)
	College or more	18032(62.07)	11017(37.93)
	unknown	6127(67.15)	2997(32.85)
	non-response	1924(55.07)	1570(44.93)
Maternal education	Middle or less	2909(58.92)	2028(41.08)
	High	22438(62.4)	13519(37.6)
	College or more	13010(61.82)	8034(38.18)
	unknown	6591(67.49)	3175(32.51)
	non-response	1845(54.88)	1517(45.12)
Substance abuse	never used	46641(62.55)	27928(37.45)
	used before	72(26.97)	195(73.03)
	in current use	80(34.78)	150(65.22)
Internet addiction	normal	42980(63.9)	24282(36.1)
	latent risk	1626(52.37)	1479(47.63)
	high risk	2187(46.54)	2512(53.46)
Single-mixed gender schools	co-education	29476(62.15)	17953(37.85)
	girls-only	7663(56.76)	5837(43.24)
	boys-only	9654(68.29)	4483(31.71)
Metro/cities/rural	metropolis	24498(62.36)	14789(37.64)
	cities	16547(62.66)	9860(37.34)
	rural	5748(61.33)	3624(38.67)
		**Mean±SD**	**Mean±SD**
Grade		3.35±1.685	3.63±1.702
Family Affluent Score		4.53±1.83	4.52±1.865
Alcohol drinking volume		0.39±1.032	0.69±1.352
Smoking volume		0.33±1.064	0.58±1.42
Strenuous physical activity		1.84±1.693	1.76±1.683
Moderate physical activity		1.79±1.607	1.75±1.622
Weight training		1.27±1.556	1.24±1.576
Self-rated sleep satisfaction		2.95±1.087	2.54±1.112
BMI		20.45±2.996	20.44±2.87
Self-rated health		3.84±0.809	3.57±0.9

**Figure 1:**
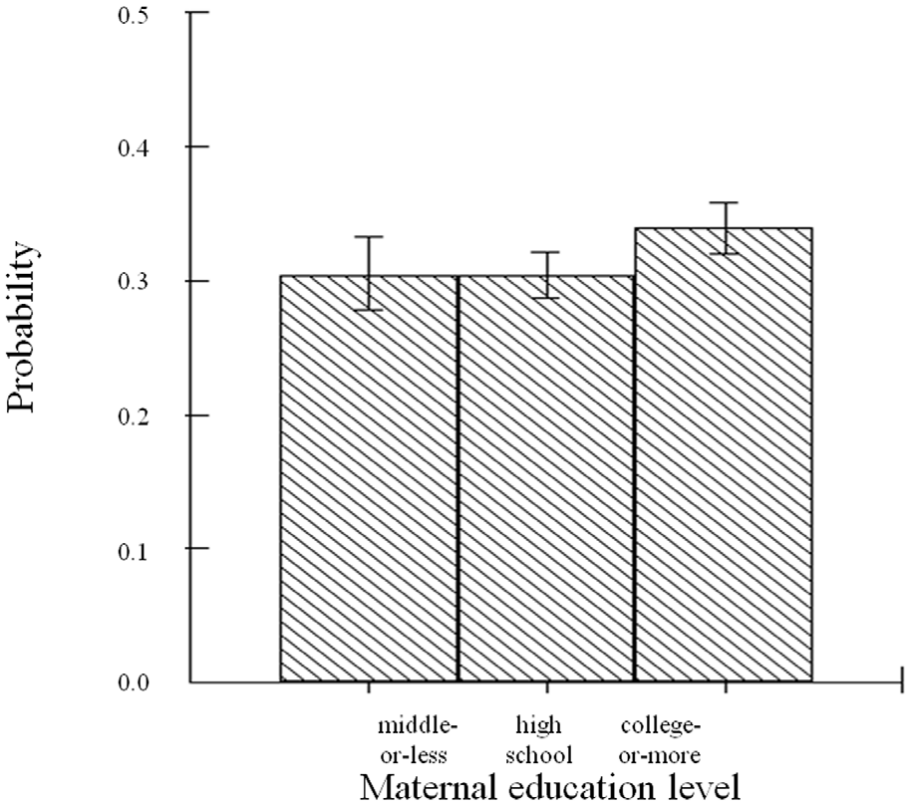
Probability of depression experience across maternal education level in male students.

Table 2 shows the results of two-level binomial logit modeling to predict the probability of depression in Korean adolescents, in two different modeling and in three different groups (both / girls / boys). Female students were more likely to experience depression than males, and increasing age was found to be associated with increasing depression experience. When we treated age as categorical variable ‘grade’, only the 12^th^ grade showed a significant difference when compared to 7^th^ grade (data not shown). The better the academic achievements the students had, the less depression experience they reported, and females showed a wider range of association of academic achievement with depression than male students. Both female and male students of higher FAS had higher rates of depression. Students without parents living together had the highest rate of depression, followed by students living with a single-parent, then students living with both. Male students living with college-educated mothers showed a statistically significant association with depression while female counterparts did not show the association (Figure 1). Several individual characteristics such as behavioral characteristics, self-rated sleep satisfaction, and BMI were significantly associated with depression. As well, attending girls-only school was more likely associated with depression in girl group. With respect to urbanicity of the school location, metropolitan areas showed significant association, in comparison to schools in rural areas; however, this relationship disappeared when parental educational attainments were modeled.

**Table 2:** Multilevel logistic regression estimates based on 2-level binomial logit model for ‘depression’ in Korean adolescents (divided by gender).

		Both		Girls		Boys	
		Model 1	Model 2	Model 1	Model 2	Model 1	Model 2
		**(N = 75066)**	**(N = 73528)**	**(N = 35454)**	**(N = 34977)**	**(N = 39612)**	**(N = 38551)**
		OR (95% CI)	OR (95% CI)	OR (95% CI)	OR (95% CI)	OR (95% CI)	OR (95% CI)
*Fixed Part*							
Intercept		1 (0.86–1.19)	**1.25 (1.05–1.73)**	**2 (1.56–2.58)**	**2.13 (1.63–2.67)**	1.06 (0.87–1.28)	1.44 (0.99–1.97)
Gender (vs. male)							
Female		**1.92 (1.83–2)**	**1.84 (1.75–1.93)**				
Age (in grade)		**1.05 (1.04–1.06)**	**1.04 (1.03–1.05)**	**1.05 (1.04–1.07)**	**1.04 (1.03–1.06)**	**1.04 (1.03–1.06)**	**1.04 (1.02–1.05)**
Academic achievement (vs. very high)							
Very low		**1.51 (1.41–1.61)**	**1.68 (1.56–1.82)**	**1.78 (1.61–1.97)**	**1.92 (1.71–2.16)**	**1.3 (1.19–1.42)**	**1.5 (1.35–1.69)**
Low		**1.26 (1.19–1.34)**	**1.4 (1.31–1.49)**	**1.44 (1.32–1.57)**	**1.56 (1.41–1.71)**	**1.12 (1.03–1.2)**	**1.28 (1.17–1.42)**
Middle		**1.18 (1.11–1.26)**	**1.25 (1.18–1.33)**	**1.3 (1.19–1.41)**	**1.35 (1.23–1.48)**	**1.09 (1.01–1.18)**	**1.19 (1.09–1.3)**
High		1.06 (0.99–1.12)	**1.09 (1.02–1.16)**	**1.14 (1.04–1.25)**	**1.17 (1.07–1.28)**	0.99 (0.92–1.08)	1.05 (0.95–1.15)
FAS		**1.02 (1.01–1.03)**	1.01 (1.00–1.02)	**1.03 (1.01–1.04)**	1.02 (1.00–1.03)	**1.02 (1.01–1.04)**	1.01 (1.00–1.02)
Parental support (vs. both parents)							
Single parent		**1.17 (1.09–1.26)**		**1.25 (1.11–1.39)**		**1.13 (1.02–1.25)**	
None		**1.32 (1.17–1.5)**		**1.49 (1.2–1.84)**		**1.25 (1.07–1.44)**	
Paternal education level (vs. middle-or-less)							
High school			**0.88 (0.81–0.96)**		**0.89 (0.8–0.99)**		**0.87 (0.78–0.98)**
College-or-more			0.96 (0.88–1.05)		0.96 (0.86–1.08)		0.97 (0.87–1.09)
Maternal education level (vs. middle-or-less)							
High school			0.99 (0.91–1.06)		0.96 (0.86–1.07)		1 (0.89–1.12)
College-or-more			**1.1 (1.02–1.2)**		1.02 (0.9–1.15)		**1.18 (1.03–1.32)**
Alcohol drinking volume	**1.14 (1.12–1.16)**		**1.15 (1.13–1.17)**	**1.17 (1.14–1.21)**	**1.18 (1.15–1.21)**	**1.12 (1.09–1.14)**	**1.13 (1.1–1.15)**
Smoking volume	**1.07 (1.05–1.08)**		**1.06 (1.04–1.08)**	**1.13 (1.1–1.17)**	**1.13 (1.09–1.17)**	**1.06 (1.04–1.08)**	**1.05 (1.03–1.08)**
Substance abuse (vs. never-used)							
Used before	**3.16 (2.25–4.41)**		**3.42 (2.19–5.35)**	**4.06 (2.03–8.64)**	**4.83 (1.99–13.8)**	**2.94 (2–4.41)**	**3.06 (1.84–5.19)**
In current use	1.5 (1–2.22)		**1.81 (1.07–3.09)**	0.87 (0.41–1.93)	1.11 (0.39–3.43)	**1.74 (1.1–2.7)**	**2.06 (1.08–3.86)**
Internet addiction (vs. normal-range use)							
Underlying danger	**1.5 (1.39–1.61)**		**1.49 (1.37–1.62)**	**1.62 (1.44–1.82)**	**1.66 (1.44–1.9)**	**1.42 (1.29–1.57)**	**1.39 (1.25–1.57)**
Danger	**1.81 (1.7–1.93)**		**1.85 (1.72–2)**	**2.06 (1.85–2.31)**	**2 (1.76–2.26)**	**1.69 (1.57–1.84)**	**1.78 (1.63–1.96)**
High level physical activity	**1.04 (1.03–1.05)**		**1.03 (1.02–1.05)**	**1.08 (1.06–1.1)**	**1.07 (1.05–1.1)**	1 (0.99–1.02)	0.99 (0.98–1.01)
Moderate level physical activity	**1.03 (1.01–1.04)**		**1.03 (1.01–1.04)**	1 (0.98–1.02)	1 (0.98–1.02)	**1.05 (1.03–1.07)**	**1.05 (1.03–1.07)**
Weight training	**1.03 (1.02–1.05)**		**1.03 (1.01–1.04)**	1.01 (0.99–1.03)	1.01 (0.98–1.03)	**1.05 (1.03–1.06)**	**1.04 (1.02–1.06)**
Self-rated sleep satisfaction	**0.78 (0.77–0.79)**		**0.79 (0.78–0.8)**	**0.77 (0.75–0.79)**	**0.78 (0.76–0.8)**	**0.79 (0.77–0.8)**	**0.8 (0.78–0.82)**
Self-rated health status	**0.75 (0.73–0.76)**		**0.74 (0.72–0.76)**	**0.72 (0.7–0.74)**	**0.72 (0.7–0.74)**	**0.77 (0.75–0.8)**	**0.76 (0.74–0.79)**
BMI	**1.006 (1.001–1.012)**		1.00 (1.00–1.01)	1.01 (1.00–1.02)	**1.011 (1.002–1.020)**	1.00 (1.00–1.01)	1.00 (0.99–1.00)
Single-mixed gender schools (vs. co-education)							
Girls-only	**0.9 (0.86–0.95)**		**0.89 (0.84–0.94)**	**0.89 (0.85–0.94)**	**0.87 (0.82–0.92)**		
Boys-only	0.99 (0.94–1.03)		0.98 (0.92–1.03)			0.98 (0.93–1.03)	0.98 (0.93–1.04)
Metro/cities/rural (vs. rural)							
Metropolitan	**1.14 (1.05–1.25)**		1.05 (0.97–1.14)	1.03 (0.93–1.14)	1.02 (0.92–1.15)	**1.16 (1.03–1.31)**	1.02 (0.89–1.16)
Cities	1 (0.95–1.05)		0.96 (0.91–1.03)	0.96 (0.89–1.03)	0.97 (0.89–1.05)	1 (0.93–1.08)	0.93 (0.85–1.02)
*Random Part*							
School level	1.02 (1.01–1.04)		1.01 (1.01–1.03)	1.01 (1–1.02)	1.01 (1–1.02)	1.02 (1.01–1.04)	1.02 (1.01–1.04)
Individual level	1		1	1	1	1	1
−2 log-likelihood	89456.634		69899.851	44042.357	35554.413	45297.664	34266.253
DIC	39.392		41.406	36.011	36.861	36.848	37.976

Model 1: adjusted variables include gender, age (in grades), self-rated academic achievement, FAS, parental support, alcohol drinking and smoking volumes, substance abuse, internet addiction, high and moderate level physical activity, weight training, self-rated sleep satisfaction and health status as individual level variables, and schools’ gender type and scale of the area school is located in as school-level variables.

Model 2: adjusted variables include those in model 1, with exception of parental support and addition of parental education level variables.

OR: odds ratio, CI: credible intervals.

Figures in bold indicate effect estimate and standard error with statistical significance.

## Discussion

This study explored significant factors related to adolescent depression in South Korea. Family economic status was positively associated with experiencing depression in both genders. As well, the highest level of maternal educational attainment was significantly associated with depression in male students. Girls had higher rates of depression than boys. Lower perceived academic achievement rating was associated with more cases of reported depression experience. In contrast, parental support had a positive influence. Unhealthy lifestyles were associated with depression experience. A protective effect on depression was found in female students attending girls-only schools.

Established findings on influence of parental education on adolescent depression have reported on association between low parental educational attainment and more likelihood of depression experience [11]–[16], and it was suggested in previous studies that the health behaviors, parenting knowledge, and sense of mastery engendered by education are of greater importance in promoting mental health in one’s children. However, our study showed opposite results that higher parental SES in this Korean sample was associated with depressed condition of their adolescent offspring. This is a notable result, and one that may be unique to the Korean cultural context, where youth have been shown to be among the least happy in developed OECD countries [17]. A previous study that explored parental locus of control (parents’ perceived power and efficacy in child-rearing situations) showed that mothers with more years of education had higher parenting self-efficacy, and insisted that education contributes to a mother’s parenting efficacy by enhancing her overall and general self-efficacy [18]. We can thus assume that mothers with higher levels of education exert more control over their children. In a society that places high priority on academic achievement, this characteristic may increase the risk for depression in adolescents. However, this finding was not significant in girls, which might have originated in girls’ more intimate relationship with same sex parents (mothers) than boys’; alternatively, it may reflect lower parental expectation for girls’ academic achievement in a patriarchal culture. Our findings call for additional research with regard to the effect of maternal educational attainment on gender difference in health outcomes. Additional research is also needed to investigate the significant inverse association between fathers’ high school educational attainment and depression in both genders.

The reason for the highest school grade, 12^th^ grade in S. Korea, to be significantly associated with adolescents’ depression can be explained by the fact that they are put under much stress while preparing for the university entrance examinations, known as “*gosambyoung*” (12^th^ grade-illness). In the same context, it is reasonable to assume that decreasing academic achievement levels might contribute to increase the likelihood of depression experience. This trend was observed in a gradual pattern over degree of academic achievement in girls while the likelihood escalated up until medium achievement level in boys. Students with a very low perceived academic achievement level reported the highest probability of experiencing depression, suggesting that Confucian culture (with its emphasis on good academic performance) may create undue mental burdens. Our suggestion is in line with a previous study where Japanese and Taiwanese students reported higher levels of parental expectation and lower levels of parental satisfaction concerning academic achievement than their American peers [19].

School context is also reported as influential on adolescent depression; a study that investigated contextual socioeconomic effects at the school level among adolescents has found school context as a risk factor for adolescents’ depressive symptoms [20]. A more recent study found school’s average socioeconomic standing as a significant school-level predictor of emotional well-being in 6^th^–10^th^ graders [21]. Although no explanation in the previous studies has been found for a negative influence of attending girls-only school on depression experience in our results, the same assumption could be applied with our finding that different inter-gender relationships at school level could be a possible influential factor in adolescent depression experience considering no strong selection mechanism between single gender school and co-education school. Beyond the well-known findings that more adolescent girls are at risk of depression than boys, the finding that girls in girls-only schools were less likely to be depressive might suggest that routine inter-gender relationships in schools do not necessarily make for good adolescent mental health. Further research on this issue is greatly encouraged. Our finding on a significant association between adolescents’ depression experience and attending schools in metropolitan areas disappeared when parental educational attainment was controlled. More highly educated parents living in metropolitan areas (composition effect) may explain the nullified association in the latter model. The inverse relationship in parental support with depressive experience, shown in living with single parent or none of the parents on comparison to living with both parents is in concordance with past studies, with a stronger association in girls than in boys [22]–[24]. As poor social support is a risk factor for recurrent depression [25], this finding is important in planning strategic measures to decrease adolescent depression.

Adolescents’ lifestyle habits investigated in our study were all significantly associated with depression. Issues of juvenile alcohol, cigarette consumption and substance abuse with psychological problems such as depression and conduct disorders have been well documented. Our results are compatible with previous reports, which differ in country and time of origin [26]–[28]. Factors related to physical health also showed different outcomes when subdivided by gender; strenuous and moderate physical activities, weight training and self-reported sleep satisfaction have some significant effect on adolescent depression experience. Questions in the survey regarding physical activities and sleep satisfaction were based only on past week recall, and we cannot assume they apply to habitual behavior. BMI was associated with adolescent depression experience only in girls. This outcome could be explained by girls being more figure-conscious than boys, and is closely related to a previous study where researchers found significant association between body image dissatisfaction and depression in girls aged 16 to 18 [29].

The FAS was first introduced in the 2^nd^ KYRBWS in 2006; investigation was made on its applicability in Korea, which in comparison to the 41 European and North American countries from which the FAS was made, has different social, economic, and cultural backgrounds [30].

Although the component of ‘total number of computers at home’ showed modest correlation, with much higher penetration rate of computers in Korea than other European countries (95% vs. 76.6% in 2006), the authors reported that FAS was in good correlation with other indicators for socioeconomic position (i.e. parental education, self-reported school achievement, perceived household economic status, and cohabitation with parents), and assured that FAS is also a useful measure of socioeconomic position in Korean adolescents.

There are several limitations in this study. Firstly the outcome variable “depression experience” was not defined by clinical diagnostic tools but was based on a single question with dichotomized answers. Many self-report questionnaire tools have been developed to assess adolescent depression: the Children’s Depression Inventory [31] (CDI), the Center for Epidemiologic Studies Depression Scale (CES-D) [32], the Depression Self-Rating Scale (DSS) [33], and the Reynolds Adolescent Depression Scale (RADS) [34]. However, as the KYRBWS survey was not specifically focused on adolescent psychological issues but rather covers a broad range of interests, only a single question on depression experience was available. This may not reflect clinical depression among the adolescents participating in the survey, and the term “depression experience” in our study should be interpreted with caution. The high prevalence of depression experience in our study (32.03% for boys and 43.96% for girls) is not true disease prevalence.

Secondly, there remains possible yet unsure bias due to the unknown data on parental education attainment (parent may not have told their children of their education attainment, or students with low parental education level may have been reluctant to report and have marked for ‘unknown’ in the questionnaire).

Thirdly, as the survey was confined to adolescents attending school, our findings did not include dropouts or habitual absentees.

Fourthly, the survey did not inquire about family history of mental problems or co-morbid psychological problems. The genetic contribution to the development of childhood and adolescent psychological problems is well established [35], and it has also been reported that adolescent depression is associated with many co-morbid psychological problems such as attention-deficit hyperactivity disorder (ADHD), conduct disorder and anxiety disorder.

Lastly, our design is cross-sectional and results cannot be used to deduce causal relationships. For example, the association between academic performance and depressive symptoms may reflect reverse causation, (i.e. depression may lead to a decline in academic performance), not the other way round (i.e. low grades and parental chiding causing depression). This is not only true for the education. While our model studies the different (cross-sectional) association, the multi-faceted aspects of the depression induce complex dynamic interactions with all the factors we studied, possibly reinforcing depression over the adolescent’s life course. This would still be the case in longitudinal studies.

In summary, we have identified several factors that were associated with adolescent depression experience in South Korea, notably between higher maternal education levels and depression experience in male offspring, and higher levels of affluence with increasing depression experience in either gender. These findings call for the attention of parents in the higher SES strata where adolescents may be hiding their mental illness from adults. Based on these original findings and the verification of some influential individual and school level factors on adolescent depression experience, the importance of an individual-, family- and school-level approach to adolescent depression should continue to be emphasized in adolescent mental health management strategies.
